# Surgical Approaches to Brachial Plexus Schwannomas: A Multidisciplinary Case Report

**DOI:** 10.1155/cro/6094879

**Published:** 2026-07-21

**Authors:** Logan A. Reed, Justin Cline, Justin Zumsteg, Karan Desai

**Affiliations:** ^1^ Department of Orthopedics, Orlando Health Jewett Orthopedic Institute, Orlando, Florida, USA; ^2^ Department of Hand Surgery, Orlando Health Jewett Orthopedic Institute, Orlando, Florida, USA

**Keywords:** brachial plexus schwannoma, clavicular osteotomy, multidisciplinary surgery, peripheral nerve sheath tumor, supraclavicular approach, transclavicular approach

## Abstract

**Background:**

Brachial plexus schwannomas are rare tumors requiring individualized surgical planning. Despite established surgical principles, the optimal approach varies substantially by tumor location, and direct comparative data across techniques remain limited.

**Cases:**

We present three cases illustrating distinct surgical strategies. A 59‐year‐old woman with foraminal and intradural extension underwent staged posterior laminectomy followed by supraclavicular resection, who recovered with no clinically significant neurologic deficits. A 69‐year‐old woman with upper trunk involvement had successful supraclavicular excision with complete symptom resolution. A 68‐year‐old man with a retroclavicular tumor required transclavicular access via clavicular osteotomy; he experienced immediate pain relief but developed osteotomy nonunion requiring revision fixation before achieving full functional recovery at 2.5 years.

**Conclusion:**

These cases demonstrate that approach selection should be driven by tumor location, anatomical extent, and intradural involvement. Careful anatomical assessment and multidisciplinary collaboration are important considerations for maximizing resection while preserving neurological function.

## 1. Introduction

Brachial plexus tumors account for approximately 5% of all peripheral nerve sheath tumors, with schwannomas and neurofibromas representing the most prevalent benign histologies [1–4]. Schwannomas arise from schwann cells, are typically encapsulated, and displace rather than infiltrate adjacent nerve fibers which can allow for safe gross‐total resection without a permanent neurological deficit [3, 5–7]. The brachial plexus presents a formidable surgical challenge due to its anatomical complexity, spanning the supraclavicular and infraclavicular regions with potential extension into the spinal canal, retroclavicular space, and thoracic inlet. Surgical approaches must therefore be selected based on precise tumor localization and may include supraclavicular, infraclavicular, transclavicular, posterior subscapular, or combined anterior–posterior exposures [8–11]. Multidisciplinary collaboration integrating neurosurgery, otolaryngology, thoracic surgery, and peripheral nerve specialists is critical to safe resection and favorable long‐term outcomes. Despite a growing literature on individual techniques, direct comparative analysis of approach selection criteria, complication profiles, and decision‐making frameworks is limited. This case series presents three patients treated through distinct surgical strategies and proposes a structured decision framework to guide approach selection in future cases.

## 2. Methods

A retrospective case series was conducted involving three patients with histologically confirmed schwannomas of the brachial plexus treated at a single tertiary referral center between 2021 and 2024. This case series was prepared in accordance with the CARE (CAse REport) guidelines. A completed CARE Checklist is provided as supporting information. Patient consent and institutional review board approval were obtained prior to study inclusion. Data were extracted from electronic medical records, including demographic information, clinical presentation, imaging findings, operative reports, pathology results, and postoperative outcomes. IRB approval and patient consent were obtained. All patients underwent preoperative magnetic resonance imaging (MRI) and electromyography (EMG) for tumor characterization and functional assessment. Intraoperative neuromonitoring was utilized in all cases. Postoperative follow‐up ranged from 1 to 30 months.

## 3. Case Presentations

### 3.1. Case 1—Combined Posterior and Supraclavicular Approach

A 59‐year‐old woman presented with several months of right‐sided neck pain radiating into her arm, accompanied by numbness, tingling, and progressive weakness. She had failed extensive nonoperative treatment including physical therapy and corticosteroid injections. Neurological examination revealed no overt motor deficits, though provocative testing over the supraclavicular region reproduced her symptoms. MRI demonstrated a T2 hyperintense, contrast‐enhancing lesion centered at the C4–C5 neural foramen with extension into the spinal canal and upper trunk of the brachial plexus. Given the lesion′s combined intradural and extraforaminal extent, a two‐stage approach was planned. In the first stage, the neurosurgery team performed a posterior C3–C6 laminectomy and fusion with intradural tumor resection (Figure 1). The second stage involved a supraclavicular approach performed jointly by otolaryngology and hand surgery for anterior plexus dissection and resection of the extraforaminal tumor component (Figure 2). Intraoperative nerve monitoring was maintained throughout both stages to preserve functional elements of the brachial plexus. Pathology confirmed a benign schwannoma. At 6‐month follow‐up, the patient had complete resolution of her neck and arm pain and full return to baseline neurological function.

**Figure 1:**
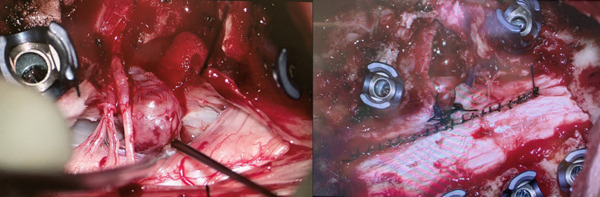
Stage one posterior cervical excision of intradural lesion for Case 1 with intraoperative images demonstrating the posterior cervical approach for resection of the intradural component of a dumbbell brachial plexus schwannoma. A C3–C6 laminectomy and fusion were performed during the first stage of the operation to access the lesion extending through the neural foramen into the spinal canal.

**Figure 2:**
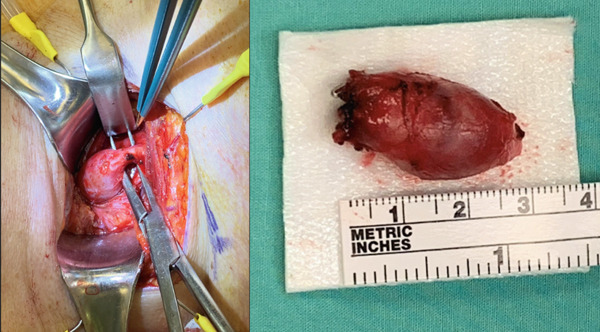
Stage two supraclavicular approach and excised mass for Case 1 with intraoperative photographs demonstrating the supraclavicular exposure utilized during the second stage of resection for Case 1. The extraforaminal component of the schwannoma involving the upper trunk of the brachial plexus was dissected and excised using microsurgical technique with intraoperative neuromonitoring.

### 3.2. Case 2—Supraclavicular Approach

A 69‐year‐old woman presented with a gradually enlarging right supraclavicular mass, stable for nearly a decade after an earlier biopsy confirmed schwannoma. Over the preceding year, she developed pain radiating to her thumb and index finger along with nocturnal paresthesias. Physical examination revealed a well‐circumscribed, mobile mass deep to the clavicle with a positive Tinel sign. Strength testing was intact in all major muscle groups, though light touch was reduced in the C5–C6 dermatomes. Updated MRI demonstrated a 3.2‐cm fusiform, encapsulated lesion arising from the posterior division of the upper trunk (Figure 3). The mass was resected through a standard supraclavicular approach involving hand and thoracic surgery. Intraoperative dissection revealed the tumor displacing surrounding nerve fibers, which were preserved using microsurgical techniques (Figure 4). No intraoperative complications occurred. Postoperatively, the patient reported marked reduction in paresthesias and full return to daily activities by her 1‐month follow‐up.

**Figure 3:**
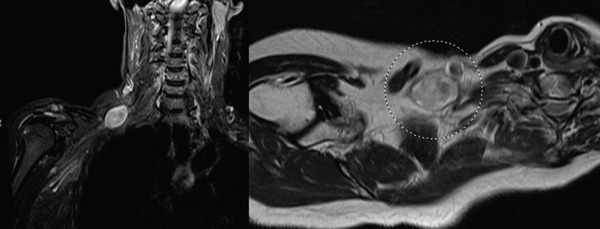
Axial and coronal MRI of Case 2 with mass on preoperative axial and coronal magnetic resonance imaging (MRI) demonstrating a well‐circumscribed, contrast‐enhancing schwannoma arising from the posterior division of the upper trunk of the brachial plexus in the supraclavicular region.

**Figure 4:**
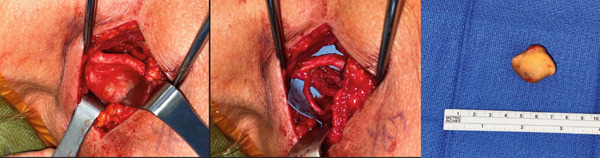
Supraclavicular approach with excision of mass for Case 2 intraoperative images demonstrating the supraclavicular surgical approach and microsurgical excision of the upper trunk schwannoma in Case 2. Surrounding fascicular structures were preserved during tumor dissection and removal.

### 3.3. Case 3—Transclavicular Approach

A 68‐year‐old man presented with a 1‐year history of worsening right upper extremity pain and weakness, including decreased grip strength and fine motor control, with difficulty using tools and numbness affecting the thumb through the ring finger. MRI demonstrated a T2 hyperintense lesion located deep in the supraclavicular fossa with caudal extension beneath the clavicle (Figure 5). Nerve conduction studies confirmed involvement of the middle trunk. Initial exploration via a supraclavicular incision revealed the mass extending into the retroclavicular space and encircling the subclavian vessels. Inadequate visualization prompted an infraclavicular extension, which still did not permit safe intrafascicular dissection. A clavicular osteotomy was therefore performed to achieve full visualization (Figure 6). Intraoperative neurophysiologic monitoring guided en bloc resection while preserving adjacent fascicles. The patient experienced immediate postoperative pain relief. However, he subsequently developed a nonunion at the osteotomy site, which required revision fixation with bone grafting. The nonunion ultimately united, and at 2.5‐year follow‐up he was fully functional with no evidence of tumor recurrence.

**Figure 5:**
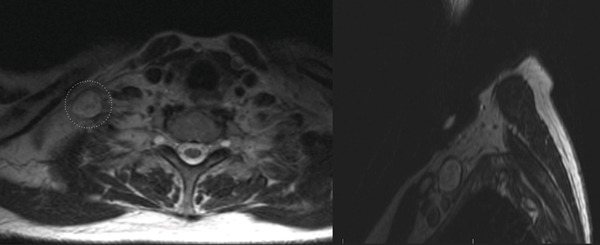
Axial and sagittal MRI of Case 3 mass on preoperative axial and sagittal MRI demonstrating a retroclavicular brachial plexus schwannoma with extension beneath the clavicle and close association with the subclavian vessels.

**Figure 6:**
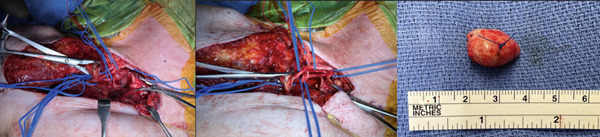
Transclavicular approach with excised mass for Case 3 with intraoperative photographs demonstrating the transclavicular surgical approach utilized to achieve adequate visualization of the retroclavicular tumor in Case 3. Clavicular osteotomy provided exposure for safe dissection and excision of the schwannoma surrounding the neurovascular structures.

## 4. Discussion

### 4.1. Novelty and Contribution

While prior series have described individual surgical approaches to brachial plexus schwannomas, few have directly compared multiple techniques within a single institutional experience and proposed an explicit decision framework. Large series by Desai et al., Jia et al., and Kim et al. establish outcome benchmarks but do not systematically address how tumor anatomy should guide approach selection [8, 12, 13]. Our series is distinctive in three respects: It demonstrates the rationale and outcome of a staged intradural–extradural resection driven by spinal canal involvement; it documents a case in which sequential supraclavicular and infraclavicular exposure proved insufficient, necessitating clavicular osteotomy; and it illustrates practical principles that may assist surgeons in selecting operative approaches based on tumor location, anatomical extent, and involvement of adjacent structures.

### 4.2. Surgical Approach Selection: Indications and Rationale

The central surgical question in managing brachial plexus schwannomas is not whether to resect but how to access the tumor safely. Considerations for surgical approach selection are determined by three anatomical variables: (1) tumor location along the plexus (roots/trunks vs. divisions/cords vs. terminal branches); (2) caudal or retroclavicular extension beneath or posterior to the clavicle; and (3) intradural or foraminal involvement. These factors governed our decision‐making in all three cases. The supraclavicular approach remains the workhorse technique for schwannomas of the upper and middle trunks. It provides direct anterior access to the roots and trunks with minimal soft tissue disruption and is ideally suited for well‐circumscribed, laterally positioned lesions without deep or caudal extension [5, 14]. In Case 2, the 3.2‐cm upper trunk lesion met precisely these criteria, and supraclavicular resection achieved complete excision without complication. The infraclavicular approach extends access to the cords and terminal branches, typically requiring mobilization of pectoral musculature, and is most useful for lesions below the clavicle not requiring proximal exposure [14]. When tumors traverse the supra and infraclavicular compartments or reside primarily in the retroclavicular space, neither approach alone provides adequate visualization. In Case 3, sequential supraclavicular and infraclavicular exposures failed to permit safe dissection around the subclavian vessels, and a clavicular osteotomy was required to achieve the necessary exposure. The transclavicular approach, though more invasive, offers the widest operative corridor for complex retroclavicular lesions [5, 9, 12]. As a fundamentally different strategy, a combined supraclavicular–infraclavicular approach without osteotomy should be attempted first, when feasible: Adequate tumor mobilization from both windows may allow delivery through either approach, preserving clavicular integrity. Osteotomy should be reserved for cases where this fails. Tumors with both intradural and extraforaminal extension which are called dumbbell lesions require a fundamentally different strategy. Single‐approach resection risks incomplete removal or neural injury at the foraminal segment. As illustrated in Case 1, a staged posterior laminectomy followed by supraclavicular resection enables comprehensive tumor removal while separating the higher risk intradural dissection from the extradural plexus work, reducing the likelihood of simultaneous complications. This combined anterior–posterior strategy has been applied successfully in dumbbell schwannomas of both the cervical and thoracic spine [4, 11]. The posterior subscapular approach represents an additional option for tumors of the proximal divisions or those requiring lateral positioning; Dubuisson et al. reported its use in over 100 patients with excellent posterior plexus access [9]. A summary of the cases is presented in Table 1.

**Table 1:** Summary of brachial plexus schwannoma cases.

Case	Symptom duration	Tumor location	Involved structure	Surgical approach	Teams involved	Follow‐up	Outcome
1	Several months	C4–C5 neural foramen with intradural and extraforaminal extension	Upper trunk/C5 root region	Staged posterior laminectomy and supraclavicular approach	Neurosurgery, otolaryngology, and hand surgery	6 months	Complete pain relief and full neurologic recovery
2	10 years with acute worsening over 1 year	Supraclavicular upper trunk lesion	Posterior division of upper trunk	Supraclavicular approach	Hand surgery and thoracic surgery	1 month	Resolution of paresthesias and return to daily activities
3	1 year	Retroclavicular lesion extending beneath clavicle	Middle trunk	Transclavicular approach with clavicular osteotomy	Hand surgery	2.5 years	Full functional recovery without recurrence

### 4.3. Comparative Analysis of Surgical Approaches

Table 2 provides a structured comparison of the principal surgical approaches, including indications, advantages, limitations, and complication profiles. This summary synthesizes data from the present series and the published literature.

**Table 2:** Comparison of surgical approaches for brachial plexus schwannomas.

Approach	Indications	Advantages	Limitations	Complications/risks
Supraclavicular	Upper/middle trunk tumors; well‐circumscribed, no caudal or intradural extension	Direct access; minimal morbidity; familiar anatomical corridor	Limited visualization of retroclavicular and lower plexus; inadequate for intradural disease	Phrenic/accessory nerve injury; lymphatic leak; Horner syndrome
Infraclavicular	Cord/terminal branch tumors below clavicle; axillary lesions	Access to cords and terminal branches; avoids clavicle	Requires pectoral muscle mobilization; limited proximal access	Vascular injury; chest wall numbness
Transclavicular (osteotomy)	Retroclavicular or deep lesions crossing supra/infraclavicular compartments; inadequate access via other routes	Maximum exposure to entire plexus; direct visualization around subclavian vessels	Most invasive; requires clavicle fixation; prolonged recovery	Nonunion (reported in ~10%–20% without rigid fixation); vascular injury; infection
Combined anterior–posterior (staged)	Tumors with both intradural and extraforaminal components; dumbbell lesions	Comprehensive exposure; allows intradural and plexus resection in planned stages	Two‐stage procedure; higher anesthetic burden; longer recovery	Cerebral spinal fluid leak; neurological deficits from cord manipulation; fusion‐related morbidity
Posterior subscapular	Proximal division tumors; lesions requiring lateral positioning	Excellent posterior plexus access; avoids anterior cervical structures	Unfamiliar exposure; limited anterior access	Periscapular muscle denervation; seroma

### 4.4. Complications: Clavicular Nonunion After Transclavicular Approach

The most clinically significant complication in our series was clavicular nonunion following transclavicular osteotomy in Case 3. This complication, while manageable, required revision fixation with bone grafting and extended the patient′s recovery. Nonunion following clavicular osteotomy is a recognized risk, with reported rates varying by fixation method and patient factors. Series employing rigid plate‐and‐screw fixation report nonunion rates in the range of 10%–20% when fixation is suboptimal, compared to lower rates with anatomic locking plate constructs [12, 15]. Risk factors include osteoporosis, smoking, inadequate immobilization, and failure to achieve stable fixation at the time of initial repair. Several technical modifications have been proposed to reduce this risk. Osteotomy site selection—preferring the middle third where cortical bone is thicker—and the use of locking compression plates rather than simple cortical screws are associated with improved union rates [16]. Postoperative immobilization protocols and avoidance of early heavy upper extremity use are also important. In our case, the initial fixation construct was revised and augmented with bone graft, ultimately achieving union at 2.5‐year follow‐up. Surgeons planning transclavicular access should counsel patients explicitly about nonunion risk, the potential need for revision, and the associated delay in functional recovery. Where an alternative approach is feasible, it should be strongly considered.

### 4.5. Role of the Multidisciplinary Team

Each case in this series was managed by a dedicated multidisciplinary team whose specific contributions directly shaped operative strategy and outcomes. In Case 1, neurosurgery led the intradural resection, whereas otolaryngology and hand surgery jointly executed the supraclavicular dissection—a division of labor that would not have been feasible by a single specialty. In Case 2, thoracic surgery was consulted for guidance on dissection around thoracic structures, whereas hand surgery performed precise microsurgical fascicle preservation during supraclavicular dissection. In Case 3, the decision to proceed with clavicular osteotomy after sequential single‐specialty attempts failed to achieve adequate exposure was made jointly intraoperatively, reflecting the kind of real‐time multidisciplinary decision‐making that cannot be replicated without co‐operative teams. These experiences align with Soltani et al. and Wilson et al., who demonstrated that team‐based management of neural sheath tumors reduces complications and improves surgical outcomes [17, 18]. Establishing dedicated brachial plexus tumor boards may further streamline preoperative planning and reduce time to surgery in this complex patient population.

## 5. Conclusion

Brachial plexus schwannomas require individualized surgical planning based on precise anatomical characterization and tumor extent. The cases presented illustrate how tumor location, retroclavicular extension, intradural involvement, and intraoperative findings may influence operative strategy and surgical exposure. Although rare, these lesions can be successfully managed through a variety of approaches tailored to the underlying anatomy. The present series highlights practical considerations for surgical planning and underscores the importance of multidisciplinary collaboration when managing complex brachial plexus tumors. Further studies involving larger patient cohorts are needed to better define optimal approach‐selection strategies and validate standardized treatment algorithms.

## Funding

No funding was received for this manuscript.

## Conflicts of Interest

The authors declare no conflicts of interest.

## Data Availability

Data sharing not applicable to this article as no datasets were generated or analyzed during the current study.
